# Zoobooth: A portable, open-source and affordable approach for repeated size measurements of live individual zooplankton

**DOI:** 10.1016/j.heliyon.2023.e15383

**Published:** 2023-04-20

**Authors:** Catharina Broch, Jan Heuschele

**Affiliations:** a Centre for Ecological and Evolutionary Synthesis, Department of Biosciences, University of Oslo, Oslo, Norway; b Section for Aquatic Biology and Toxicology, Department of Biosciences, University of Oslo, Oslo, Norway; c Centre for Biogeochemistry in the Anthropocene, The Faculty of Mathematics and Natural Sciences, University of Oslo, Oslo, Norway

**Keywords:** Body size, Repeated measurements, Handling risk, Stress, Video analysis, Zooplankton, *Daphnia*, Open hardware

## Abstract

Repeated size measurements of individual animals are valuable data for many research questions, but it is often hard to obtain without causing stress or damage to the animal. We developed a video-based approach called Zoobooth to size individual zooplankton, which involves a low risk of handling accidents and stress. Here we describe the process of assembling the instrument we used to acquire video recordings of single zooplankton and the procedure to obtain size estimates from the recorded videos. Our setup produces accurate size estimates for *Daphnia magna* (correlation to manual measurements = 0.97), and was also tested with other zooplankton species. Zoobooth is especially advantageous when one needs size measurements of live, individual mesozooplankton. The device is small, portable, and comprised of very affordable and readily available components. It can easily be modified for other purposes, such as studies of coloration or behavior of micro-and macro-plankton. We share all the files to build and use Zoobooth.

## Graphical abstract

**Figure:**
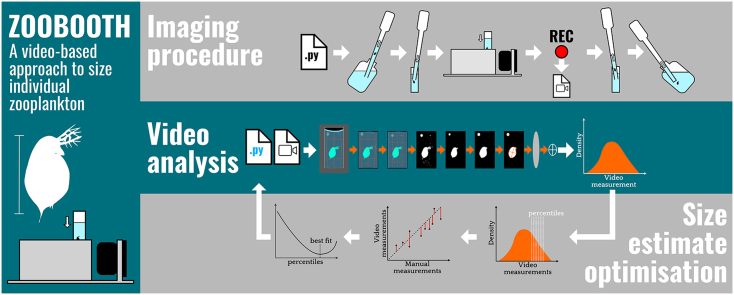

• We developed a video-based approach to repeatedly size individual zooplankton, which involves a low risk of handling accidents and stress.
• The device is small, portable, and comprised of very affordable and readily available components
• We include a simple procedure to optimize the parameter settings for different zooplankton species

## 1 Introduction

Body size is a crucial and defining feature of most organisms. For pelagic organisms in the ocean, individual size is the most important characterizing trait because it links to metabolic rates, clearance rates, swimming speeds, and sensory ranges [1]. In limnic pelagic ecosystems, the size structure of zooplankton communities is often a good indicator of the system's trophic structure, as explained by the Size-Efficiency Hypothesis, which encapsulates the community-structuring role of size-dependent competition and predation [2]. Moreover, size is a crucial trait that shapes the ecological niches of phytoplankton because many ecophysiological traits correlate with cell size [3].

Although the size of an organism theoretically is a simple trait to measure, it can be challenging to obtain in practice. It is, for instance, usually much easier to take body size measurements of non-moving individuals, which requires that the targeted individual is immobilized or killed before measurement. In plankton research, the small sizes and the usually large number of study subjects make the acquisition of size data a very labor-intensive task. For these and other reasons, several instruments have been developed that increase the efficiency of obtaining plankton size data. One example is the Optical Plankton Counter. This instrument provides a quantitative method based on optical detection and automates the collection of abundance and size data of zooplankton in marine [4] and fresh waters [5]. Two other automated methods are the FlowCam and ZooScan analysis systems. These use imaging techniques and image analysis to collect data on shape, size, and other physical properties from organisms in micro- and macroplankton samples [6,7]. In the Optical Plankton Counter and the FlowCam, samples are passed in front of a detection device (photodiode, camera), which then registers the number and size of each organism. The image obtained by the FlowCam can further be used to classify the organisms. In ZooScan a sample of immobilized zooplankton is placed on a scanning device, and the high-resolution image is then processed to obtain information about the organisms’ size, their numbers, and classification.

The abovementioned instruments provide highly efficient and sophisticated methods for obtaining data on plankton community abundance and diversity. They are thus suitable for studies on species distribution and community structure in natural aquatic ecosystems. However, for laboratory studies on a single or a few plankton species, these instruments are often less attractive, although they have been successfully used for such purposes as well. For example, Kessler & Lampert [8] used an Optical Plankton Counter to obtain reliable population counts and population size distribution data on monospecific cultures of *Daphnia hyalina*. However, a major drawback with the instruments mentioned above is that they do not easily let you follow individual specimens, which can be a requirement in laboratory studies where one wants to associate an individual's size with other physiological traits or life-history parameters. Also, the required fixation of the samples makes these methods unsuitable for projects where one needs to collect size data from live specimens repeatedly.

A traditional and frequently used method to obtain individual size measurements of zooplankton is to measure the specimen manually on a glass slide under a microscope, either directly or from photographs [[9], [10], [11], [12], [13], [14], [15]]. This technique provides precise data, but is relatively time-demanding, requires immobile specimen, and introduces observer subjectivity. The observer subjectivity can be evaded by substituting manual measurements of photographed glass slides with image analysis, as done in e.g., Archarya et al. [16] and Kyle et al. [17], and is an approach that is facilitated by the R package zooimage [18]. This imaging method requires that the specimens are immobile on the glass slide, normally done by trapping the individual zooplankton in a flat water droplet. However, this approach invariably involves delicate handling of the specimen, which increases the risks of accidents and potentially introduces stress to the organism.

Handling-induced stress has been reported to affect growth in catfish *Clarias fuscus* [19] and reproduction in the striped trumpeter *Latris lineata* [20]. In Daphnia, the effects of handling are, to our knowledge, not thoroughly documented. However, Rousseaux et al. [21] found no significant effects of frequent pipetting and microscope measurements on the clutch size or the age at reproduction for the first two clutches in *Daphnia magna*. Although this suggests that *D. magna* is quite robust to experimental handling, such as trapping the individual in a droplet of water, frequent handling will always increase the risk of accidents and potentially pose significant stress to more delicate species. Moreover, the traditional method entails that the specimen must be taken out of its experimental conditions (e.g., temperature and light). These issues might be of little concern for studies where one takes a single or a few body size measurements of robust zooplankton. However, they become essential in studies requiring repeated measures or testing subtle differences in environmental conditions on delicate species.

Growth is a process that typically requires many repeated measurements to describe, as an organism's body size typically changes due to development, resource acquisition, and senescence. This is the case for most mesozooplankton, such as *D. magna*, which has an adult size that is 3–5 times its newborn length. Moreover, the growth process is susceptible to environmental conditions (e.g., resource levels and temperature) and can also vary significantly between populations [15,22]. These are relatively well-established insights. However, more information is needed on the degree of intraspecific differences in growth for zooplankton of the same population or the same clone. In a recent project, we wanted to address this aspect of plankton development and follow the growth of many Daphnia individuals across their entire lifespan. For this purpose, we required a method that would provide us with accurate size measurements that involved a low risk of handling accidents and stress to the animal. In addition, we needed the technique to be realized in a portable setup so that we could carry the instrument between the culture rooms of the experiment.

The published methods for tracking live Daphnia did not apply to our project for different reasons. The Multi-DaphTrack [23] and the millifluidic chip-based system described in Huang et al. [24] are primarily suited for tracking behavior, while the Daphniatox [25] and the image analysis approach of live Daphnia described in Færøvig et al. [26] are best suited for obtaining population data. The automated imaging approach described in Heuschele et al. [27] is developed for sizing meiobenthic animals and is less suitable for pelagic free-swimming animals since the filming is done from below, and the high-throughput microtiter well-plate system described in Duckworth et al. [28] seems to require a long image acquisition time.

Since none of the available methods were suited for our purpose, we developed a new method and analysis technique that provided a way of obtaining accurate size measurements of individual zooplankton involving little handling risks and stress to the animal, and where the method was time-efficient, simple to apply, and could be realized in a portable setup. Our setup, called Zoobooth, uses affordable and readily available components (Raspberry Pi, Pi NoIR camera module). Raspberry Pis are affordable single-board computers that are increasingly used in the biological sciences and that have proven to be very useful for a whole range of applications, both in the lab and in the field [29]. The program and analysis scripts are written in widely used, open-source programming languages (Python, R). The Zoobooth has proven itself highly effective in one large, long-term Daphnia project and in several research experiments on smaller scales. This paper first describes the setup and procedure for length measurements of *D. magna* with the Zoobooth, and then assesses how our method performs on five other microcrustacean zooplankton.

## 2 Materials and methods

**Fig. 1:**
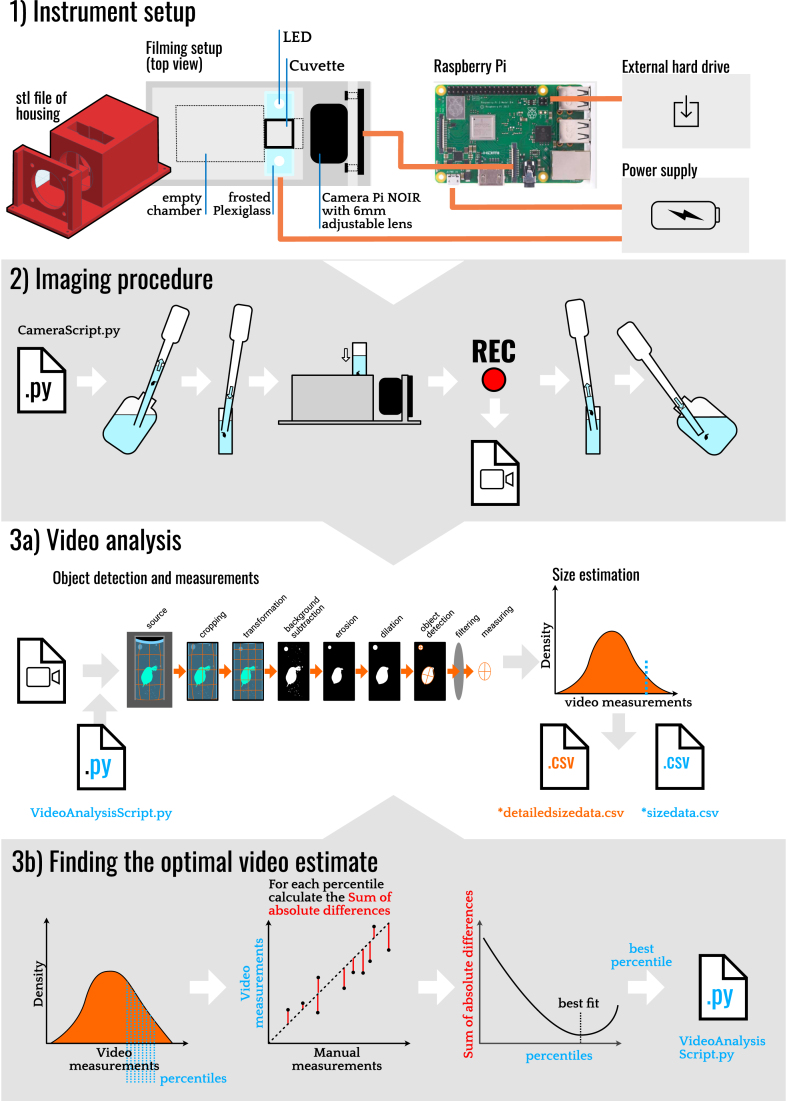
Schematic overview of the Zoobooth approach. 1) The different setup components and the connection between them. 2.) The imaging procedure starts with pipetting the animal into a cuvette, recording the movie, and placing the animal back into the culturing vessel. 3a) Overview of the frame manipulation and analysis steps in the video analysis Script and the two resulting CSV files with size data. 3b) Steps involved in determining the optimal percentile for each species which is then used in the video analysis script. More details about the approach are described in the text.

We present the Zoobooth approach in three different steps, as illustrated in Fig. 1. First, we describe the design and build of the setup for capturing videos of single individuals of zooplankton. In the second step, we introduce the imaging procedure that we applied to create the video files. In the last step, we describe how we analyze these video files to obtain accurate size estimates of the specimens.

### 2.1 Instrument setup

Our setup consists of a camera connected to a single-board computer that can film individual zooplankton in a small, illuminated container. We also added a power bank and a hard drive for more storage and placed all components in a portable plastic case.

We mounted the camera and the light source in a plastic housing that we printed with a 3D printer with standard polylactic acid plastic (Fig. 1, step 1). The housing accommodates one standard disposable cuvette (outer dimensions of 1.2 × 1.2 × 4.5 cm), which can hold the measured animal. We share the setup's shapefile in the supporting information (CameraHouse.stl). Alternatively, the camera and lights could also be held in place using laser-cut frames or even LEGO pieces.

We used the Raspberry Pi NOIR camera (v2) with a C mount, 6 mm adjustable-focus lens, and mounted it sidewards facing the cuvette. The camera was connected to a small single-board computer (Raspberry Pi model 3 B+) and controlled by a simple python script (CameraScript.py; supporting information). To prevent stray light from the camera indicator LED that could influence the organism's behavior while filming, we disabled it in the settings of the Raspbian OS. On both sides of the filming booth, we placed two white LEDs on the top facing downwards into two frosted Plexiglas cuboids in order to have even illumination of the chamber. The empty compartment on the rear side of the cuvette minimizes light reflection from the back and provides a dark-field image. We connected a standard 7″ touchscreen monitor in a display case to the Raspberry Pi to be able to change the sample identifier in the script and start the recording using the graphical user interface. We used an external hard drive (1 TB) for data storage. The entire system can be powered with a rechargeable power bank or a conventional power source. If the power bank is not providing enough power (indicated by a lightning bolt appearing on the monitor screen), you should use a conventional power source when possible. We fitted all components of the setup in a small instrument case, which served the purpose of protection, storage, and easy transportation. In total, the material needed to assemble the setup sum up to 330 euros; however, the essential items (Raspberry pi, sd card, and camera) are nowadays available for around 130 euros.

During filming, we placed the animals in cuvettes. We used standard macro cuvettes (internal depth 12 mm) for the adult *D. magna* and half-micro cuvettes (internal depth 4 mm) for juvenile *D. magna* and the other zooplankton species to ensure that the animals remained in the camera's focal plane. In both cases, we only filled the cuvettes with medium slightly above the camera's field of view so that the animals could not swim out of the camera's view during recording.

Because our camera lens introduced a slight barrel distortion that could influence the measurements, we determined the camera matrix to correct the distortion in the video analysis. For this, we filmed a miniature checkerboard, extracted some video frames, and then used the calibX.py script from OpenCV to calculate the matrix (Open Source Computer Vision; https://docs.opencv.org/master/dc/dbb/tutorial_py_calibration.html). The script results in four files (dist.out, mtx.out, newcameramtx.out, roi.out; supporting information), which are used during the video analysis to correct for the barrel distortion of the lens system. The calculation of the camera matrix needs to be done only once when the setup is mounted, and the camera focus and zoom are fixed. The four files should be placed in a separate subfolder named “lenscalibration” within the directory of the video analysis script.

### 2.2 Imaging procedure

The imaging procedure itself is straightforward (Fig. 1, step 2). You pipette the targeted individual from its culture jar to the cuvette and place it in the allocated slot in the plastic housing. Next, you execute the camera script using the terminal interface (python CameraScript.py), which records and saves a video file directly to the external hard drive. During the recording process, a real-time image of the filming booth will appear on the screen monitor, allowing you to evaluate whether the individual was visible at its full length in the camera's field of view for at least a couple of seconds. The camera script we use (CameraScript.py; supporting information) is a python script that uses the picamera package (v1.13, https://picamera.readthedocs.io/en/release-1.13/index.html) to interface with the camera. The script creates a video file, in the h264 format (resolution 1080 × 768 pixels), of a set length, which in our case was 30 s long, but that can easily be changed. We found that for our setup and use, the optimal camera settings were an ISO of 200, a sharpness of 50, a framerate of 26, and a switched-off exposure mode, while for the other settings, we relied on the default values. The long recording time was necessary to ensure that the Daphnia swam freely through the field of view and was not connected to the sides of the cuvette. One could potentially optimize the setup by using a bigger container and moving the camera further away to increase the depth of field.

An additional feature of the camera script is that you can add identifying information to the recording by changing the parameter values in the first lines of the script. Here we used it to identify the targeted individual and treatment (for example, temperature treatment, the individual's age, and ID). This input is then incorporated into the filename of the recorded video.

To summarize, the imaging procedure is as follows: you start by pipetting the targeted individual from its culture container to the cuvette, then place the cuvette in the allocated slot in the camera house, change the relevant identifying information in the camera script, execute the script, and while the 30 s video is recording, you can evaluate from the live preview whether the animal was captured in the video. If the movie is ok - in this context, a moving animal in the field of view for more than 3 s - you take the cuvette out of the camera house and pipette the individual back to its culture container. If not, you repeat the recording. With this procedure, you have obtained one video file of the individual that is saved to the external hard drive, which can be identified by the video file name.

### 2.3 Video analysis

We analyze the videos with a custom python script (VideoAnalysisScript.py; supporting information) using code for object detection from the Open-Source Computer Vision Library (OpenCV, Version 3.0). In short, our script takes a video file and extracts a size measurement of the targeted object from each picture frame in the video when the object meets a set of defined criteria (Fig. 1, step 3a). The criteria, such as brightness, minimum size, and position, are necessary to ensure that the object a) is an individual zooplankton, b) in focus, and c) free swimming. Thus, from each video recording of one individual zooplankton, the video analysis script collects a large set of size estimates that will be of different values due to the free rotation of the animal in the filming booth.

In more detail, our video analysis script is used as follows. When started via the terminal, the program begins with a dropdown menu where the user can choose the target species, which loads species-specific filter criteria as outlined in Table 1, which are used to reduce the number of false detections. The user then chooses the folder containing the files that need to be analyzed and can provide a suffix to the output filename. Before any size estimates are extracted from each video, each frame undergoes a series of image manipulations, as depicted in Fig. 1. First, the frame is cropped to the inner area of the cuvette. To correct for the slight barrel distortion of our lens system, the image is then transformed using the initially calculated camera matrix with the function cv2.undistort (OpenCV). Afterward, the program converts the transformed image to grayscale and determines its overall sharpness using the functions within cv2.Laplacian (OpenCV), which is used later in the filtering process. Next, the program subtracts the background using K-nearest neighbors-based segmentation algorithm (cv2.createBackgroundSubtractorKNN; OpenCV). Then the image is eroded to remove small particles in the water and noise in the background, as well as diminish delicate structures of the animal, such as the spine and antennae (cv2.erode, OpenCV). At last, the image is dilated to compensate for the previous step's erosion, bringing the object's main body back to its original width and length (cv2.dilate, OpenCV).

**Table 1:** Parameter values for the different genus to filter the detected objects during the video analysis. For all groups we used a requirement of a minimum sharpness of 1.8, and a minimum distance of the edge of 10 pixels.

Genera	Minimum Area (px)	Maximum Area (px)	Minimum Length/Width ratio	Maximum Length/Width ratio	Minimum brightness	Optimal percentile
Daphnia^a^	1500	4000	1.2	1.7	30	93
Eucyclops & Mesocyclops	290	850	1.3	1.9	20	90
Diaphanosoma	300	2500	1.5	2.5	20	100
Heterocope	600	4000	1.6	2.2	20	100
Polyphemus	250	1000	1.0	1.7	20	98
Scapholebrius	250	1800	1.0	1.7	20	96

a The values for Daphnia are also used as default values in case no species is chosen from the dropdown menu.

After these image manipulations, all particles in the image are detected using a contour-finding algorithm (cv2.findContours, OpenCV). The largest object is selected and tested against a set of species-specific criteria (filtering process), which are initially chosen from the dropdown menu at the program's start. The criteria are i) the size range of the object (MinArea and MaxArea), ii) the length-to-width ratio of the object (MinLWratio and MaxLWratio), iii) the brightness of the object (MinBrightness), iv) the sharpness of the object, and v) the distance between the center of the object and the edge of the cuvette. These filtering criteria are used to ensure that objects are indeed zooplankton individuals; as different species differ in size, shape, and other morphological features the criteria need to be adapted to each taxon to improve the size estimation. Additional species and their filter criteria can be easily added to the script. If the detected object meets the criteria defined in the filtering process, the program fits an ellipse and saves the major axis as the object's length and the minor axis as the object's width. The size measurements made in pixels are converted to millimeters using a conversion factor that we estimated once the camera lens was initially fixed in the setup.

In the supporting information, we share two examples that depict how the program fits an ellipse to each video frame that fulfills the set of criteria described above (ObjectDetection_AdultDaphnia.mp4, ObjectDetection_JuvenileDaphnia.mp4, Electronic appendix S4). Thus, the video analysis produces a sequence of size estimates from each video corresponding to the sequence of filtered video frames (depicted as a density distribution in Fig. 1). The complete record of the filtered size estimates from each video is stored in a text file named *.detailedsizedata.csv. Linked to each video size estimate, the program saves the video's file name, which serves as the data identifier. The duration of the analysis depends on the length of the video file and the computing power. Still, even on a low-powered laptop, a 30-s video is processed within 11 s.

As an additional feature of the video analysis, we programmed it to save the first 19 video frames from which measurements are taken as separate picture files. We added this feature because it provides an easy way of manually inspecting the analysis process, and one can also validate the video size estimates by taking manual measurements of the images of the imaged individual.

To analyze a collection of video files, you store the collection of videos in the same folder, choose the folder, and the program will sequentially load each video file, execute the video analysis, and sequentially save the results into the text file.

### 2.4 Finding the optimal video size estimate

The size analysis of one video of one individual produces a sequence of length estimates. The length estimates will vary primarily due to the free three-dimensional rotation of the animal in the filming booth but also because the video analysis sometimes fails to identify the correct outline of the individual. Consequently, we expect considerable variability in the estimated values and that the actual length of the individual is captured in the collection of length estimates. Immediately, one might consider calculating the mean or median value from the collection of measurements as an estimate for the individual's length. However, the length measurements are not normally distributed around the true value for different reasons. Many of the measurements will be smaller than the individual's true size because the individual, in many cases, will not swim completely parallel to the camera. In fewer cases, when the length estimate is larger than the individual's true size, this would, in the case of *D. magna*, primarily be caused by the image manipulation processes failing to erode the Daphnia's spine and antenna. We, therefore, included a statistical approach to find the best descriptive statistic from the distribution of length measurements produced by the video analysis that corresponded better to the individual's actual length than the mean or median value would.

For this, we prepared a set of assessment data on *D. magna* and five other crustacean zooplankton species. We manually collected measured length estimates in addition to the length estimates produced by the video approach. Details about these data are presented in the assessment section below. Then we compared the manually obtained estimates with the percentiles from the distribution of video estimates. The complete analysis is shown in the R markdown document, which we share in the supporting information (FindingOptimalVideoEstimate.pdf), and which we can summarize as follows (Fig. 1, step 3b). For each individual in the data set, we calculate the 50th to the 100th percentile from the distribution of length estimates produced by the video analysis. Then we find the percentile from the video distribution that best corresponds to the manual length estimate across all data points in the dataset. This we do by calculating the sum of the absolute difference between the manual estimate and one specific percentile from the distribution of video estimates (e.g., the 75 percentile). Next, we simply picked the percentile that gave the lowest absolute difference to the manual measurement and chose this as the optimal video estimate. For *D. magna,* the percentile from the distribution of video length estimates that best corresponded to the manual measurements was the 93rd percentile. However, for the other plankton species, the best fit was provided by a different percentile (Table 1, Fig. 3). The result could also be sensitive to specifics of the instrument design, e.g. the volume of the filming booth, and we therefore recommend that this step of the procedure is re-assessed when a new set-up is mounted.

Having found the percentile from the distribution of video estimates that best corresponded to the manual estimates for each taxon, we add the value as a fixed parameter (Optimalpercentile, see also Table 1) in the script. With this information included, we made the video program produce an additional text file named *sizedata.csv which only included the best-fit-percentile as the estimate for each recorded individual. Here, we also included a count of the number of video frames that supports the size estimate. Although we could have created this file from the complete record of size estimates saved in *.detailedsizedata.csv, we found it convenient to immediately get the processed size estimates.

### 2.5 Assessment

We collected two groups of data that we used to assess the size measurements on live individual zooplankton obtained with the Zoobooth setup and video analysis technique. The first group of data is from individuals of *D. magna* cultured in the lab, and the second group of data is from a diverse set of microcrustacean freshwater zooplankton collected from the field. The size data on *D. magna* was collected in two different ways. One subset of the data is from individuals of *D. magna* that we specifically cultured for the assessment, and the second subset is extracted from the results of a large experiment where we applied Zoobooth to obtain size measurements of *D. magna*.

For the subset of size estimates of *D. magna* that were cultured for the assessment, 39 individuals were reared under normal conditions at 20 °C and then measured at six different ages (5 individuals at age 1, 3, 9, 12 and 15 days, and 9 individuals at age 50). The individuals were first recorded with the Zoobooth instrument to obtain length estimates by video analysis as described above, and then the length of each individual was manually determined from microscope photographs (Leica Microsystems; Leica Application Suite) by measuring the Daphnia's length from the base of the spine to the top of the head.

For the subset of size estimates of *D. magna* that were extracted from the results of a large experiment, we used the initial frames that are saved by the video analysis to obtain the manual measurements using ImageJ. The subset contains 156 data points associated with 10 individuals of *D. magna* (kept at 20 °C) from the age of 0–108 days. We restricted the manual measurements to pictures where the animal was oriented in such a way that we could measure the length of the individual as we did it for the first group of assessment data (base of the spine to the top of head). Thus, for each recorded video, we have a collection of picture samples together with the manual and automated size estimates of the targeted individual.

The second group of assessment data is from a diverse set of microcrustacean zooplankton that we collected from three freshwater localities in the vicinity of Oslo, Norway (59°58′13.8″N 10°37′24.7″E, 59°58′27.4″N 10°37′51.4″E, 59°58′15.2″N 10°37′42.3″E). The collection includes individuals of a calanoid copepod (*Heterocope appendiculata*), two cyclopoid copepods (*Eucyclops serrulatus*, *Mesocyclops leuckarti*), and three species of cladocerans (*Diaphanosoma brachyurum*, *Polyphemus pediculus, Scapholebrius mucronata*). The video size estimates were obtained using our video setup, and the manual measurements were measured from microscope photographs using ImageJ.

**Fig. 2:**
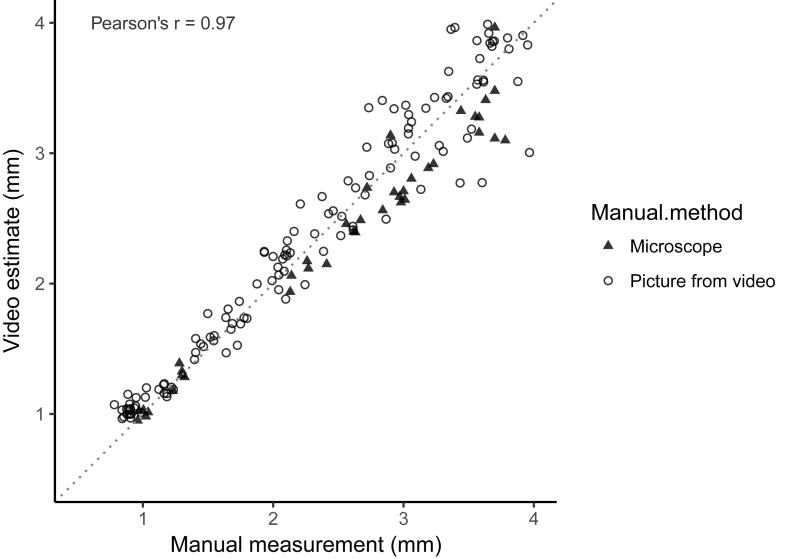
A correlation plot between the length estimates of the *Daphnia magna* in our assessment data. The dotted line gives the 1:1 correlation between the two measurement methods. The video estimate is the 94th percentile from the distribution of length estimates produced by the video analysis. The manual measurements were either made from microscope photographs (filled triangles) or from video pictures (open circles).

We then determined the percentile from the distribution of video length estimates that best corresponded to the manually measured length of the six different taxa in our assessment data. For *D. magna* we found that the best statistic was the 94th percentile. The correlation between this video estimate and the manual measurements is depicted in Fig. 2. The video method overestimates the length of the newborn Daphnia slightly, but otherwise, there is a strong linear correlation between the estimates from the manual measurements and the video analyses (Pearson's correlation coefficient = 0.966). The absolute difference between the length estimates is on average 0.10 mm for individuals smaller than 2 mm (average 1.27 mm), 0.18 mm for individuals between 2 and 3 mm (average 2.49 mm), and 0.32 mm for individuals larger than 3 mm (average 3.70), which give an average relative percentage difference (average difference/average size x 100) of 8.03, 7.49 and 8.62%, respectively.

**Fig. 3:**
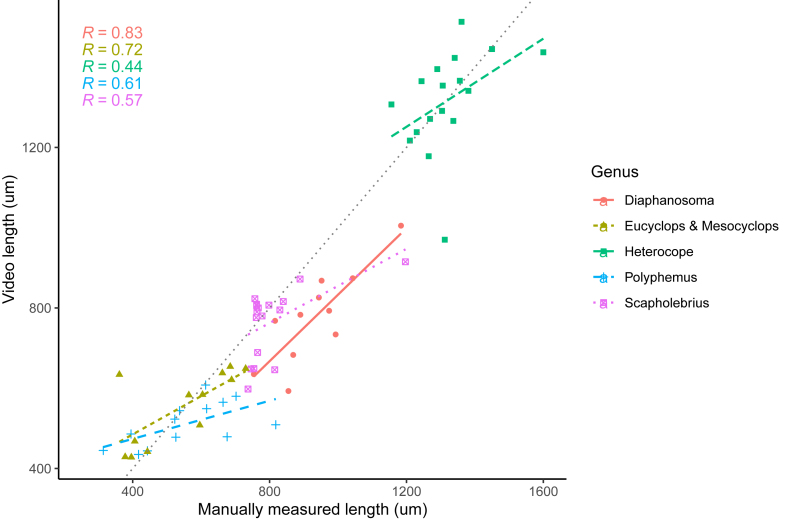
Relationship between manual length measurements and the Zoobooth measurements for different field sampled zooplankton genera. Eucyclops and Mesocyclops were combined due to similarity in appearance.

The comparison of manual and Zoobooth estimates for a set of diverse zooplankton species shows differences in the estimation accuracy between the species (Fig. 3). Within species, the fit between manual and automated sizes varied a lot, while pooled over all species there was a good agreement between both methods (R^2^ = 93%). For Diaphanosoma the video measurement underestimated the size of the individuals. An investigation of the saved images revealed that the object detection process failed to include the head of the animal due to the stark differences in body transparency. However, the underestimation was consistent throughout the different sizes, therefore a fitted linear model still had an R^2^ of 0.83. The second-best fit was achieved for the cyclopoid copepods (R^2^ of 0.72). For the other species, the copepod Heterocope, and the two cladocerans Polyphemus and Scapholebrius the relationship between manual and automated measurements was worse.

## 3 Discussion

We developed a new method to obtain repeated size measurements of individual *D. magna* that minimizes handling risk and stress to the animal. The Zoobooth method has proven itself highly effective with Daphnia and shows potential with other microcrustaceans. The setup has been applied in two master projects on Daphnia [30,31] and one long-term experiment. In the long-term experiment, we experienced a very low rate of accidental killings considering the high frequency of measurements and the duration of the experiment (one year). Only 7 of 320 Daphnia were killed during the year-long experiment with on average 26 recordings per animal. There is good reason to believe that the handling involved with the size measurements caused little stress to the animals, as they had a life span comparable to healthy *D. magna*. For example, the four clones of *D. magna* reared at 15 °C in this experiment had a mean lifespan of 113.8, 75.3, 74.2 and 132.5 days (sd of 40.1, 48.0, 23.5 and 39.3 days respectively; Broch personal communication), which is above the reported lifespan of other *Daphnia* species in e.g. Armitage and Landau (1982) [32] and Orcutt and Porter (1983) [33].

The size estimates from the video analysis showed a strong linear correlation with the manual estimates of size, and the relative percentage difference between the size estimates was on average also low. Still, it is indisputable that the accuracy of the Zoobooth method is lower than what one can achieve from manual measurements. But we consider that our method provides an accuracy that is adequate for many purposes, especially when the data is used to parameterize growth models (e.g. fitting a Von Bertalanffy growth function). Our method also offers an easy way to validate the size estimates by using picture files that are automatically saved during the video analysis.

While we originally designed the system to obtain size measurements of *D. magna*, our approach can be extended and adjusted for many other purposes and species as well. With only few changes to the filtering criteria in the script, we managed to use the setup with different zooplankton species. Our test revealed that Zoobooth in its current form works best for size estimates of species that have a morphology similar to Daphnia, or a simple spherical shape and evenly colored body. For species with uneven body transparency and prominent appendages, the script and potentially also the size of the container need further adjustments (Fig. 3).

We designed our approach to be small, portable, and simple to use. The complete setup fits in an instrument case that is 33 × 28 cm wide and has a total weight of only 2.5 kg. This allows us to move the instrument to where the animals are kept for the experiment, rather than having to repeatedly move the animals out of their experimental conditions to the location of the instrument. This is advantageous for controlled laboratory experiments, and it opens the possibility of bringing the instrument along in the field. It arguably requires some technical skills to assemble the setup and some insight into programming to adjust the video analysis script. However, once set up, Zoobooth is easy to use.

The method is quite time efficient. It takes on average 1 min to obtain one video recording of an individual. The video recording lasts 30 s, and the user spends the same time changing the ID information in the camera script and piping the individual from its culture jar to the cuvette and back again. Depending on the swimming behavior of the target species, a shorter video duration might also be sufficient to acquire accurate size measurements.

Our setup is low-cost. We spent 30 euros on the camera, 100 euros on all the equipment related to the Raspberry Pi, and approximately 230 euros on the rest of the equipment, which gives a total price of 330 euros (354 US dollars). The prices might vary between countries. We believe this is affordable for most scientists involved in research, teaching, and science communication projects.

### 3.1 Comparison with alternative approaches

The Zoobooth approach offers a cheap, robust, accurate, and efficient way of obtaining repeated size measurements of individual zooplankton that cannot easily be copied by the alternative approaches described in the literature. The Multi-DaphTrack [23] and the millifluidic chip-based system [24] were designed for analyzing Daphnia behavior. Although it is probably possible to redesign these approaches to obtain repeated estimates of zooplankton size while keeping them under different exposure scenarios (for example, different temperature regimes), we cannot see how this could easily be done. Moreover, the setup presented in Chevalier et al. [23] is not mobile as it requires to be associated with a cooling system. Also, neither of the two approaches seem practicable for repeated measurements due to their cell chip design.

Further, when considering the approaches described in Færøvig et al. [26] and Hader et al. [25], they seem like efficient approaches to obtain population counts from samples of live zooplankton as well as estimates of the population size distribution. Still, we consider the accuracy of individual size measurements to be considerably lower than what we achieve with Zoobooth since the imaging systems had a lower resolution and assessed larger sample volumes. Of all the available approaches that describe techniques for imaging live mesozooplankton, the procedure described in Duckworth et al. [28] is the one that most resembles the approach we present in this paper. Duckworth and colleagues present a technique for obtaining size measurements of individual aquatic invertebrates with the aid of the spheroid counter Cell [3]iMager, which is a commercially available machine based on scanning technology. One strength of their method is that you can measure many single individuals simultaneously since the Cell [3]iMager is built to scan well plates. However, the duration of the imaging processes is, in return, considerably longer. Duckworth et al. [28] write in their protocol that the well plates with the animals were placed in the spheroid counter for 2 h of scanning. It is unclear whether this is a strict requirement of the method or whether the image acquisition time was chosen without a strong preference for time efficiency since the imaging process anyways is automated. Long acquisition times might become an issue in cases where one wants to make repeated measurements because it could entail a notable change in the experimental conditions for the animals.

Another area for improvement for the approach by Duckworth et al., which is acknowledged in the article, is the accuracy of the size measurements. They suspect that the relatively low accuracy is caused by the free three-dimensional orientation of the animals in the well, which they had not found a way to account for. This is also an issue that Bruijning et al. [34] did not address in their particle tracking program TRACKDEM, which is designed to collect population counts and size distributions of live animals from videos in R. In their assessment of TRACKDEM, they used 35 individuals of *D. magna* to evaluate the accuracy of the size estimates by comparing manual measurements with the estimates from the video analysis, which estimate particle size by calculating the median size in pixels over all frames in the video. To evaluate the correlation between the manual measurement and the video estimates, they report R^2^-values of 0.80 and 0.68. For comparison, if we compute the R^2^ from our assessment data by fitting a linear model with the manual measurements as the independent variable and the video estimate as the dependent variable, we get an R^2^-value of 0.93. Thus, our approach of optimizing the settings for the video analysis (step 3a and 3b) offers a significant improvement compared to the existing methods of obtaining size estimates from images of moving, non-spherical organisms.

When we tested our measuring device with other zooplankton species, we find that species that resemble Daphnia or have a uniformly elliptic shape without strong appendages, like the copepods, give good size estimates without manipulating the image manipulation steps in the script. The weaker correlation between manual and video measurements for Polyphemus, Scapholebrius and Heterocope can be partially explained by differences in body shape and appearance, like bigger appendages and partially transparent body parts. For example, the stronger antenna of Polyphemus would need an adjustment of the image manipulation steps (such as erosion and dilation steps) prior to measuring the animal, which was outside the scope of our study. Similarly, species identification and filtering could be aided in the future using deep learning algorithms that experts could train to only measure from suitable frames.

Compared to commercial solutions, Zoobooth is also considerably cheaper, easily repairable, and can be easily modified in the future to accommodate different-sized containers and organisms. It is thus in the spirit of the current open electronics and hardware movement [35]. Compared to other Zooplankton tracking approaches, it is also portable and can be used in the field.

### 3.2 Relevancy and potential for other applications

Somatic growth is an essential process in all organisms because of its association with life-history processes such as reproduction and survival. The size of an organism can also be linked to the organism's position in the food web, to swimming speeds, clearance rates, metabolic rates, and ecophysiological traits [[1], [2], [3]]. Size is typically a highly plastic trait that varies during ontogeny and with environmental conditions. It is thus evident that variability in size among individuals of either environmental or genetic origin can be an important factor in determining individual fitness. Individual differences in traits affecting fitness can also translate into significant effects at the population level [[36], [37], [38], [39]]. Still, our understanding of the ecological consequences of individual variability in fitness-related traits is limited because population ecology typically focuses on population averages [39]. Accentuating this, Fontana et al. [40] state that individual variability in traits is one of the least understood aspects of microbial communities. This is assertedly also true for communities of marine and freshwater plankton. The Zoobooth system provides a method to obtain such individual data, which is difficult to obtain with existing methods.

Besides size measurements, the analysis script could easily be modified to study the coloration of zooplankton. This can be relevant in studies of parasite infection dynamics or when studying responses to environmental stressors such as UV. The Zoobooth approach for imaging pelagic mesozooplankton could also be adjusted for different species of macro- or microplankton by changing the camera setup or substituting the camera lens.

The Zoobooth can also be a valuable tool for communicating and teaching aquatic science. As a demonstration, we have shared a time-lapse movie in the supporting information (DaphniaTimelapse.mp4) that depicts the growth of individual Daphnia at different temperatures. Such visual demonstrations are an appealing and convincing way of presenting biological processes compared to data figures. Furthermore, in an era where science and society are becoming increasingly dependent on computer technology, we believe that the Zoobooth and similar approaches could be a starting point for teaching computer literacy and programming to students in aquatic sciences. For example, a course could include a group project where students are given the task to first print and assemble the setup, and in a second step to develop or optimize the analysis procedure to answer a given or self-defined research question. This type of teaching would potentially incite strong student engagement, which enhances student performance and the student's problem-solving abilities [41,42]. Additionally, it would be a good way to introduce students and researchers to the possibilities of open electronics and open science, a growing movement with multiple benefits for researchers and science in general [35].

We developed an approach for obtaining repeated size measurements of live individual zooplankton that involves a low risk of handling accidents and stress to the animal. It includes an imaging system, automated object detection, and a simple statistical procedure to extract the best size estimate for single individuals. Our approach provides size data with higher accuracy than similar alternative approaches and is at the same time relatively simple to use, portable, and very affordable. The method has been applied successfully in one long-term experiment that collected individual-based data on a large number of *Daphnia magna*, and here we have also demonstrated that the method can be adjusted to work with other microcrustacean zooplankton as well. The Zoobooth provides an efficient approach for obtaining individual-based data on planktonic organisms, which is relevant to advance our insight in aquatic community ecology and population dynamics.

## Author contribution statement

Catharina Broch; Jan Heuschele: Conceived and designed the experiments; Performed the experiments; Analyzed and interpreted the data; Contributed reagents, materials, analysis tools or data; Wrote the paper.

## Data availability statement

Data included in article/supp. material/referenced in article.

## Declaration of competing interest

The authors declare that they have no known competing financial interests or personal relationships that could have appeared to influence the work reported in this paper.
