# Supplementary material for: Zoobooth: A portable, open-source and affordable approach for repeated size measurements of live individual zooplankton
Source: Heliyon. 2023 Apr 20;9(5):e15383. doi: 10.1016/j.heliyon.2023.e15383 (PMC10160350; doi:10.1016/j.heliyon.2023.e15383)
Supplement: Multimedia component 1 [file mmc1.zip › SizeAnalysis_Bogstadvannet.html]

Bogstadvannet Zooplankton


# Bogstadvannet Zooplankton

#### Catharina Broch and Jan Heuschele

#### 2021-09-23

```
library(tidyverse)
```

**Read in files**

```
manual.measurements <- read.csv("avg_size_manual.csv", header = T)
colnames(manual.measurements) <- c("Row", "ID", "Manual_Length_um")

video.size.Diaph <- read.csv("Cladocera_Diaphanosoma.csv", header=T)
video.size.Poly <- read.csv("Cladocera_Polyphemus.csv", header=T)
video.size.Scapho <- read.csv("Cladocera_Scapholebrius.csv", header=T)
video.size.Cop1 <- read.csv("Copepoda_Copepod1.csv", header=T)
video.size.Hetero <- read.csv("Copepoda_Heterocope.csv", header=T)

video.measurements <- rbind(video.size.Diaph, video.size.Poly, video.size.Scapho, video.size.Cop1, video.size.Hetero)
colnames(video.measurements) <- c("Movie", "Video_Length_mm", "Video_Width_mm", "Frame_count")

video.detailed.size.Diaph <- read.csv("Cladocera_Diaphanosoma_DetailedSizedata.csv", header=T)
video.detailed.size.Poly <- read.csv("Cladocera_Polyphemus_DetailedSizedata.csv", header=T)
video.detailed.size.Scapho <- read.csv("Cladocera_Scapholebrius_DetailedSizedata.csv", header=T)
video.detailed.size.Cop1 <- read.csv("Copepoda_Copepod1_DetailedSizedata.csv", header=T)
video.detailed.size.Hetero <- read.csv("Copepoda_Heterocope_DetailedSizedata.csv", header=T)

video.measurements.detailed <- rbind(video.detailed.size.Diaph, video.detailed.size.Poly, video.detailed.size.Scapho, video.detailed.size.Cop1, video.detailed.size.Hetero)
colnames(video.measurements.detailed) <- c("Movie", "Video_Length_mm", "Video_Width_mm")
```

### Video result: Summary statistic

**Extract specimen info**

```
head(video.measurements)
```

```
##                                                                    Movie
## 1 Taxa_Cladocera_Type_Diaphanosoma_brachyurum_ID_74_20200702-205951.h264
## 2 Taxa_Cladocera_Type_Diaphanosoma_brachyurum_ID_73_20200702-205312.h264
## 3 Taxa_Cladocera_Type_Diaphanosoma_brachyurum_ID_68_20200702-203944.h264
## 4 Taxa_Cladocera_Type_Diaphanosoma_brachyurum_ID_67_20200702-203020.h264
## 5 Taxa_Cladocera_Type_Diaphanosoma_brachyurum_ID_70_20200702-204623.h264
## 6 Taxa_Cladocera_Type_Diaphanosoma_brachyurum_ID_77_20200702-210805.h264
##   Video_Length_mm Video_Width_mm Frame_count
## 1       0.6349423     0.01086728          17
## 2       0.7676172     0.01313806          46
## 3       0.7932492     0.01357677          80
## 4       0.8680801     0.01485752         156
## 5       0.5934769     0.01015759          27
## 6       0.8257538     0.01413309         136
```

```
refined.video.measurements <- separate(video.measurements, Movie, c("out1", "Taxa", "out2", "Genus", "Species", "out3","ID", "out4"), sep = "_",  remove = TRUE, convert = TRUE) %>% select(-c(out1, out2, out3, out4)) 

head(refined.video.measurements)
```

```
##        Taxa        Genus    Species ID Video_Length_mm Video_Width_mm
## 1 Cladocera Diaphanosoma brachyurum 74       0.6349423     0.01086728
## 2 Cladocera Diaphanosoma brachyurum 73       0.7676172     0.01313806
## 3 Cladocera Diaphanosoma brachyurum 68       0.7932492     0.01357677
## 4 Cladocera Diaphanosoma brachyurum 67       0.8680801     0.01485752
## 5 Cladocera Diaphanosoma brachyurum 70       0.5934769     0.01015759
## 6 Cladocera Diaphanosoma brachyurum 77       0.8257538     0.01413309
##   Frame_count
## 1          17
## 2          46
## 3          80
## 4         156
## 5          27
## 6         136
```

**Merge data**

```
bogstad.zooplankton <- refined.video.measurements %>% left_join(manual.measurements[,c("ID", "Manual_Length_um")])
head(bogstad.zooplankton)
```

```
##        Taxa        Genus    Species ID Video_Length_mm Video_Width_mm
## 1 Cladocera Diaphanosoma brachyurum 74       0.6349423     0.01086728
## 2 Cladocera Diaphanosoma brachyurum 73       0.7676172     0.01313806
## 3 Cladocera Diaphanosoma brachyurum 68       0.7932492     0.01357677
## 4 Cladocera Diaphanosoma brachyurum 67       0.8680801     0.01485752
## 5 Cladocera Diaphanosoma brachyurum 70       0.5934769     0.01015759
## 6 Cladocera Diaphanosoma brachyurum 77       0.8257538     0.01413309
##   Frame_count Manual_Length_um
## 1          17              754
## 2          46              816
## 3          80              974
## 4         156              952
## 5          27              855
## 6         136              944
```

```
bogstad.zooplankton$Video_Length_um <- round(bogstad.zooplankton$Video_Length_mm * 1000, 0)
```

**Plot**

```
library(ggpubr)
bogstad.zooplankton %>% 
  mutate(Genus = recode(Genus,  "Copepod" = "Eucyclops & Mesocyclops")) %>%
  ggplot(aes(Manual_Length_um, Video_Length_um, 
             linetype = Genus,
             shape = Genus, 
             col = Genus)) + 
  geom_point() + 
  geom_abline(slope = 1, 
              linetype=3, 
              col="gray50") + 
  theme_classic() + 
  scale_x_continuous("Manually measured length (um)", 
                     breaks = seq(400,2400,400)) + 
  scale_y_continuous("Video length (um)", 
                     breaks = seq(400,2400,400)) +
  geom_smooth(method ="lm", se = FALSE, size=0.7) + 
  stat_cor(aes(label = ..r.label..)) ->
  p1

p1
```

```
ggsave("Figure3.pdf", p1, width = 7.5, height = 5)
ggsave("Figure3.png", p1, width = 7.5, height = 5)
```

```
ggplot(subset(bogstad.zooplankton, Genus =="Copepod"), aes(Manual_Length_um, Video_Length_um)) + geom_point(col="grey50") + geom_text(aes(label=ID)) + geom_abline(slope = 1, linetype=3, col="gray50") + theme_bw() + ggtitle("Copepod") + ylim(350,750) + xlim(350,750)
```

```
ggplot(subset(bogstad.zooplankton, Genus =="Diaphanosoma"), aes(Manual_Length_um, Video_Length_um)) + geom_point(col="grey50") + geom_text(aes(label=ID)) + geom_abline(slope = 1, linetype=3, col="gray50") + theme_bw() + ggtitle("Diaphanosoma") + ylim(550,1200) + xlim(550,1200)
```

```
ggplot(subset(bogstad.zooplankton, Genus =="Heterocope"), aes(Manual_Length_um, Video_Length_um)) + geom_point(col="grey50") + geom_text(aes(label=ID)) + geom_abline(slope = 1, linetype=3, col="gray50") + theme_bw() + ggtitle("Heterocope") + ylim(950,1600) + xlim(950,1600)
```

```
ggplot(subset(bogstad.zooplankton, Genus =="Polyphemus"), aes(Manual_Length_um, Video_Length_um)) + geom_point(col="grey50") + geom_text(aes(label=ID)) + geom_abline(slope = 1, linetype=3, col="gray50") + theme_bw() + ggtitle("Polyphemus") + ylim(300,850) + xlim(300,850)
```

```
ggplot(subset(bogstad.zooplankton, Genus =="Scapholebrius"), aes(Manual_Length_um, Video_Length_um)) + geom_point(col="grey50") + geom_text(aes(label=ID)) + geom_abline(slope = 1, linetype=3, col="gray50") + theme_bw() + ggtitle("Scapholebrius") + ylim(550,1200) + xlim(550,1200)
```

**Correlation**

```
bogstad.zooplankton <- na.omit(bogstad.zooplankton)

cor(bogstad.zooplankton[, c("Manual_Length_um", "Video_Length_um")], method="pearson")
```

```
##                  Manual_Length_um Video_Length_um
## Manual_Length_um        1.0000000       0.9364396
## Video_Length_um         0.9364396       1.0000000
```

### Video result: Detailed data

**Extract specimen info**

```
head(video.measurements.detailed)
```

```
##                                                                    Movie
## 1 Taxa_Cladocera_Type_Diaphanosoma_brachyurum_ID_74_20200702-205951.h264
## 2 Taxa_Cladocera_Type_Diaphanosoma_brachyurum_ID_74_20200702-205951.h264
## 3 Taxa_Cladocera_Type_Diaphanosoma_brachyurum_ID_74_20200702-205951.h264
## 4 Taxa_Cladocera_Type_Diaphanosoma_brachyurum_ID_74_20200702-205951.h264
## 5 Taxa_Cladocera_Type_Diaphanosoma_brachyurum_ID_74_20200702-205951.h264
## 6 Taxa_Cladocera_Type_Diaphanosoma_brachyurum_ID_74_20200702-205951.h264
##   Video_Length_mm Video_Width_mm
## 1       0.6266135     0.01072473
## 2       0.5971059     0.01021970
## 3       0.6349423     0.01086728
## 4       0.5952875     0.01018857
## 5       0.6023110     0.01030878
## 6       0.6090069     0.01042339
```

```
refined.video.measurements.detailed <- separate(video.measurements.detailed, Movie, c("out1", "Taxa", "out2", "Genus", "Species", "out3","ID", "out4"), sep = "_",  remove = TRUE, convert = TRUE) %>% select(-c(out1, out2, out3, out4)) 

head(refined.video.measurements.detailed)
```

```
##        Taxa        Genus    Species ID Video_Length_mm Video_Width_mm
## 1 Cladocera Diaphanosoma brachyurum 74       0.6266135     0.01072473
## 2 Cladocera Diaphanosoma brachyurum 74       0.5971059     0.01021970
## 3 Cladocera Diaphanosoma brachyurum 74       0.6349423     0.01086728
## 4 Cladocera Diaphanosoma brachyurum 74       0.5952875     0.01018857
## 5 Cladocera Diaphanosoma brachyurum 74       0.6023110     0.01030878
## 6 Cladocera Diaphanosoma brachyurum 74       0.6090069     0.01042339
```

**Merge data**

```
bogstad.zooplankton.detailed <- refined.video.measurements.detailed %>% left_join(manual.measurements[,c("ID", "Manual_Length_um")])
head(bogstad.zooplankton.detailed)
```

```
##        Taxa        Genus    Species ID Video_Length_mm Video_Width_mm
## 1 Cladocera Diaphanosoma brachyurum 74       0.6266135     0.01072473
## 2 Cladocera Diaphanosoma brachyurum 74       0.5971059     0.01021970
## 3 Cladocera Diaphanosoma brachyurum 74       0.6349423     0.01086728
## 4 Cladocera Diaphanosoma brachyurum 74       0.5952875     0.01018857
## 5 Cladocera Diaphanosoma brachyurum 74       0.6023110     0.01030878
## 6 Cladocera Diaphanosoma brachyurum 74       0.6090069     0.01042339
##   Manual_Length_um
## 1              754
## 2              754
## 3              754
## 4              754
## 5              754
## 6              754
```

```
bogstad.zooplankton.detailed$Video_Length_um <- round(bogstad.zooplankton.detailed$Video_Length_mm * 1000, 0)
```

```
ggplot(bogstad.zooplankton.detailed, aes(Manual_Length_um, Video_Length_um)) + geom_point(alpha = 0.5) +
  geom_abline(slope = 1, linetype=2, col="limegreen") + facet_wrap(~Genus)
```
