# Supplementary material for: Zoobooth: A portable, open-source and affordable approach for repeated size measurements of live individual zooplankton
Source: Heliyon. 2023 Apr 20;9(5):e15383. doi: 10.1016/j.heliyon.2023.e15383 (PMC10160350; doi:10.1016/j.heliyon.2023.e15383)
Supplement: Multimedia component 1 [file mmc1.zip › FindingOptimalVideoEstimate.html]

FindingOptimalVideoEstimate.utf8.md


### Supporting information

#### Accompanying the article *Zoobooth: A portable, open-source and affordable approach for repeated size measurements of individual zooplankton*

Catharina Broch and Jan Heuschele

## Finding the optimal video estimate

**Dependencies**

```
library(tidyverse)
library(readxl)
```

#### Data

**Manual measurements**

As we describe in the article, we collected two groups of data on *Daphnia magna* that we used to assess how the video length estimates produced by the Zoobooth technique compare to manual measurements. In the first group of assessment data we made the manual measurements from microscope photographs (example picture A below), and in the second group we made the manual measurements from one of the 19 still pictures that the video program saves during the video analysis process (example picture B below).

We read in the the manual measurements from the following three files:

```
data.manual.lotta_1 <- read_excel("Zoobooth_Leica_Lotta_1.xlsx")
data.manual.lotta_2 <- read_excel("Zoobooth_Leica_Lotta_2.xlsx")
data.manual.lotta_3 <- read.table("Ecovar_20C_Lotta_Manual measurements.txt", header=TRUE)
```

**Complete record of estimates from video analysis**

The complete record of the length estimates from the video analysis, associated to the *Daphnia* individuals in our assessment data, are compiled in the three files we read in next.

```
detailed.video.lotta_1 <- read_csv("Zoobooth_Video_Lotta_1_DetailedSizedata.csv")
detailed.video.lotta_2 <- read_csv("Zoobooth_Video_Lotta_2_DetailedSizedata.csv")
detailed.video.lotta_3 <- read.csv("Ecovar_20C_Lotta_DetailedSizedata.csv")
```

Below you see how the structure of the detailed data file produced by the video analysis looks like. In column two and three are the estimates of length and width from the detected object in each filtered video frame. And column one gives the name of the video file from which the size estimates were extracted from.

```
head(detailed.video.lotta_3)
```

```
##                                                  Movie   Length      Width
## 1 EV_Temp20_CloneLotta_age27_ID_2_20170422-112750.h264 3.136376 0.05368028
## 2 EV_Temp20_CloneLotta_age27_ID_2_20170422-112750.h264 2.876874 0.04923881
## 3 EV_Temp20_CloneLotta_age27_ID_2_20170422-112750.h264 2.726388 0.04666319
## 4 EV_Temp20_CloneLotta_age27_ID_2_20170422-112750.h264 2.880707 0.04930440
## 5 EV_Temp20_CloneLotta_age27_ID_2_20170422-112750.h264 3.085105 0.05280276
## 6 EV_Temp20_CloneLotta_age27_ID_2_20170422-112750.h264 3.358478 0.05748165
```

The name of the video file contains the information that identifies the data points. For our purpose here, we are only interested in the individual’s age and ID, and we thus only extract that information from the text string in the first column.

```
refined.detailed_Lotta_1 <- separate(detailed.video.lotta_1, Movie, c("out1", "out2", "out3", "out4", "out5", "Age","out6", "ID", "out7"), sep = "_",  remove = TRUE, convert = TRUE) %>% select(-c(out1, out2, out3, out4, out5, out6, out7)) %>%   separate(Age, c("out","Age"), sep = 3, remove = TRUE, convert = TRUE) %>% select(-c(out))

refined.detailed_Lotta_2 <- separate(detailed.video.lotta_2, Movie, c("out1", "out2", "out3", "out4", "out5", "Age","out6", "ID", "out7"), sep = "_",  remove = TRUE, convert = TRUE) %>% select(-c(out1, out2, out3, out4, out5, out6, out7)) %>%   separate(Age, c("out","Age"), sep = 3, remove = TRUE, convert = TRUE) %>% select(-c(out))

refined.detailed_Lotta_3 <- separate(detailed.video.lotta_3, Movie, c("out1","out2","out3","Age","out4", "ID", "out7"), sep = "_",  remove = TRUE, convert = TRUE) %>% select(-c(out1, out2, out3, out4, out7)) %>%   separate(Age, c("out","Age"), sep = 3, remove = TRUE, convert = TRUE) %>% select(-c(out))
```

The data files with the video size estimates then look like this:

```
head(refined.detailed_Lotta_3)
```

```
##   Age ID   Length      Width
## 1  27  2 3.136376 0.05368028
## 2  27  2 2.876874 0.04923881
## 3  27  2 2.726388 0.04666319
## 4  27  2 2.880707 0.04930440
## 5  27  2 3.085105 0.05280276
## 6  27  2 3.358478 0.05748165
```

**Merge video estimates with manual measurements**

Next, we merge the estimates from the video analysis with the manual measurements.

```
Lotta_1 <- refined.detailed_Lotta_1 %>% left_join(data.manual.lotta_1[,c("Age", "ID", "size_mm")])
Lotta_1$Manual.method <- "Microscope"

Lotta_2 <- refined.detailed_Lotta_2 %>% left_join(data.manual.lotta_2[,c("Age", "ID", "size_mm")])
Lotta_2$Manual.method <- "Microscope"

Lotta_3 <- refined.detailed_Lotta_3 %>% left_join(data.manual.lotta_3[,c("Age", "ID", "size_mm")])
Lotta_3$Manual.method <- "Video picture frame"
Lotta_3 <- na.omit(Lotta_3)

detailed.data.combined <- rbind(Lotta_1, Lotta_2, Lotta_3)
```

The panel plot below depicts how the complete record of length estimates from the video analysis compare to the manually made length measurements. The dashed green line gives the 1:1 line between the two variables. The plot clearly shows that the video analysis produces many estimates that are smaller than the individual’s true size, and fewer that are larger.

```
ggplot(detailed.data.combined, aes(size_mm, Length, color = Age)) + geom_point(alpha = 0.5) +
  geom_abline(slope = 1, linetype=2, col="limegreen") + facet_wrap(~Manual.method) +
  ylab("Video estimates (mm) ") + 
  xlab("Manual measurement (mm)") + theme_bw() + xlim(c(0, 7)) + ylim(c(0, 7))
```

#### Analysis: Finding the optimal video estimate

We want to find the percentile from the distribution of size estimates from the video analysis (example histogram below) that best correspond to the size estimate from the manual measurement.

In the plot below we show the distribution of video size estimates from one observation unit, a *Daphnia* 50 days of age, with the ID number = 1, and with a manually measured body length of 3.44 mm (dashed vertical line).

```
subset.detailed.data <- subset(detailed.data.combined, Age == 50 & ID == 1 & size_mm == 3.44)

ggplot(subset.detailed.data, aes(Length)) + geom_histogram() + theme_bw() + geom_vline(xintercept = 3.44, col="violetred", linetype=2)
```

We start the analysis by grouping all video size estimates by each observation unit. Then we calculate the percentiles from the distribution of video size estimates for each of the observation units. For illustration, in the code that follows we have extracted the 75th percentile from all distributions and show how we evaluate how this video size estimate corresponds to the manual measurement. In the complete analysis we extract all percentiles between 50-100.

```
percentile.i <- 75

sort.data <- detailed.data.combined %>% 
    group_by(Age, ID, size_mm) %>%
    summarise(percentlength = quantile(Length, percentile.i*0.01))
```

The figure below depicts how the 75th percentile from the distribution of video size estimates (percentlength) corresponds to the manually measured lengths across all observation units in the assessment.

```
ggplot(sort.data, aes(size_mm, percentlength)) + geom_point() + geom_abline(slope = 1, linetype=2, col="limegreen") + theme_bw() + xlim(c(0.5,5)) + ylim(c(0.5,5)) + annotate(geom="text", x=0.6, y=5, label=paste("Percentile = ",percentile.i), color="gray20", hjust=0, size=4)
```

Next, we calculate how much the estimate from the video analysis (percentlength) differs from the manual measurement. The result for the 75th percentile is presented below.

```
sort.data$difference <- sort.data$percentlength - sort.data$size_mm

ggplot(sort.data, aes(1:length(difference), difference)) + geom_point() + geom_hline(yintercept = 0, linetype=2, col="violetred3") + theme_bw() + xlab("Index") + annotate(geom="text", x=5, y=-1.5, label=paste("Percentile = ",percentile.i), color="gray20", hjust=0, size=4)
```

Then, we transform these differences to absolute values and calculate the sum of the absolute differences. In this way we get a measure of how much the 75th percentile from the distribution of video size estimates differs from the manual measurement.

```
sort.data$absolute.difference <- abs(sort.data$difference)
sum(sort.data$absolute.difference)
```

```
## [1] 63.81547
```

This we do for all percentiles between 50 and 100, and then we find the percentile that gives the lowest sum of absolute differences. This percentile we choose as the optimal size estimate from the distribution of video size estimates.

**Complete analysis**

```
statistics.table <- data.frame()

for(i in seq(50,100, 1)) {
  sort.data <- detailed.data.combined %>% 
    group_by(ID, Age, size_mm) %>%

    summarise(percentlength = quantile(Length, i*0.01))
  
    sort.data$difference <- sort.data$percentlength - sort.data$size_mm
    sort.data$absolute.difference <- abs(sort.data$difference)
    sum.absdiff <- sum(sort.data$absolute.difference)
    
  statistics.table <- rbind(statistics.table, c(i, sum.absdiff))
}

names(statistics.table) <- c("Percentile", "Sum.Absolute.Difference")
```

```
statistics.table %>%   
  group_by(Percentile) %>% 
  slice(which.min(Sum.Absolute.Difference)) %>% 
  ggplot(aes(x = Percentile, y = Sum.Absolute.Difference)) + theme_bw() +
  geom_point() + geom_vline(xintercept = 94, linetype = 2, col=4)
```

```
OptimalPercentile <- statistics.table %>% 
  slice(which.min(Sum.Absolute.Difference)) 

OptimalPercentile <- simplify(OptimalPercentile[1,1])
OptimalPercentile
```

```
## [1] 94
```

In our case, it was the 94th percentile that gave the smallest difference between the manual measurement and the video estimate, and we therefore choose this as the optimal estimate from the video analysis.
