# Supplementary figures and images for: Zoobooth: A portable, open-source and affordable approach for repeated size measurements of live individual zooplankton
Source: Heliyon. 2023 Apr 20;9(5):e15383. doi: 10.1016/j.heliyon.2023.e15383 (PMC10160350; doi:10.1016/j.heliyon.2023.e15383)

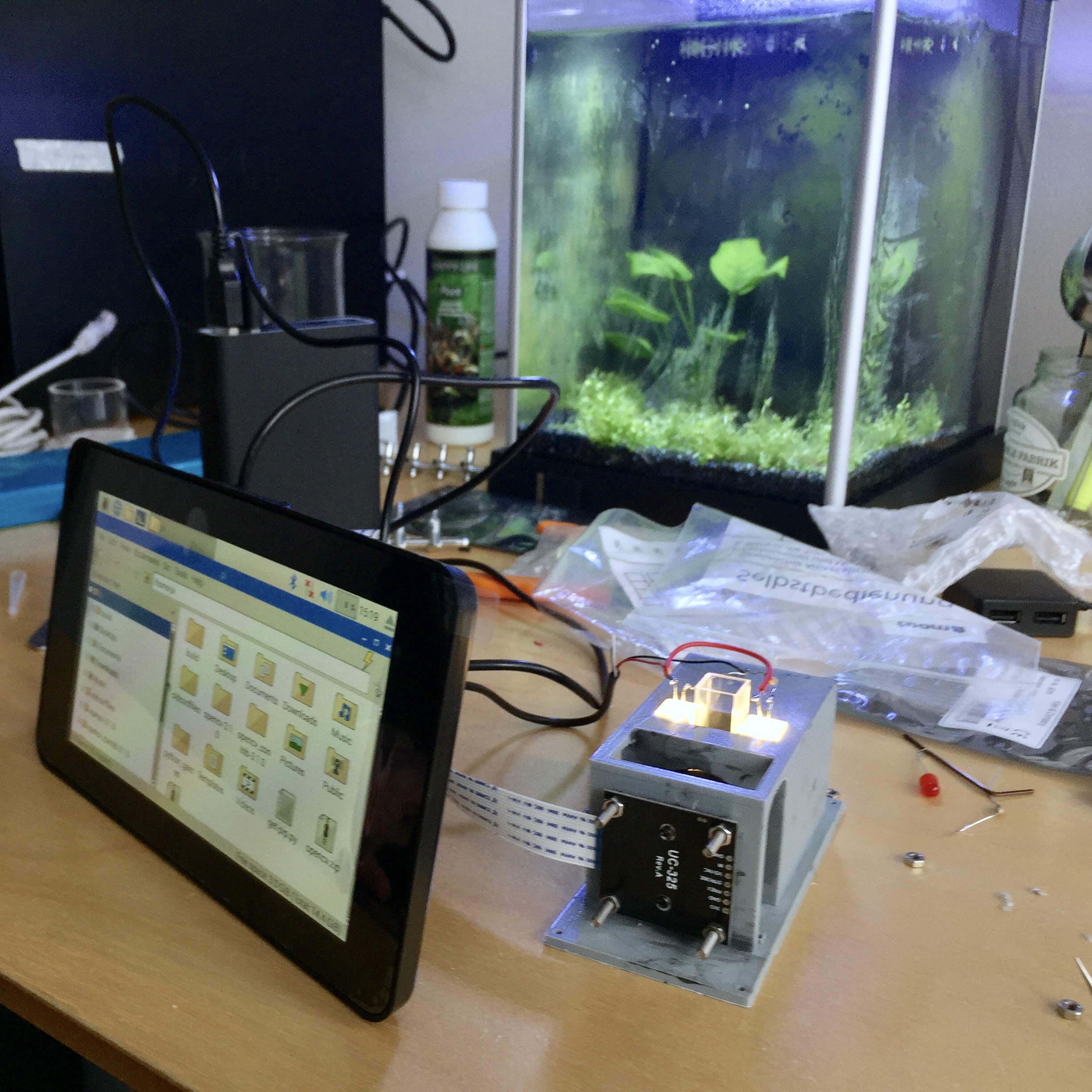

Supplement: Multimedia component 1 [file mmc1.zip › photoofsetup2.JPG]

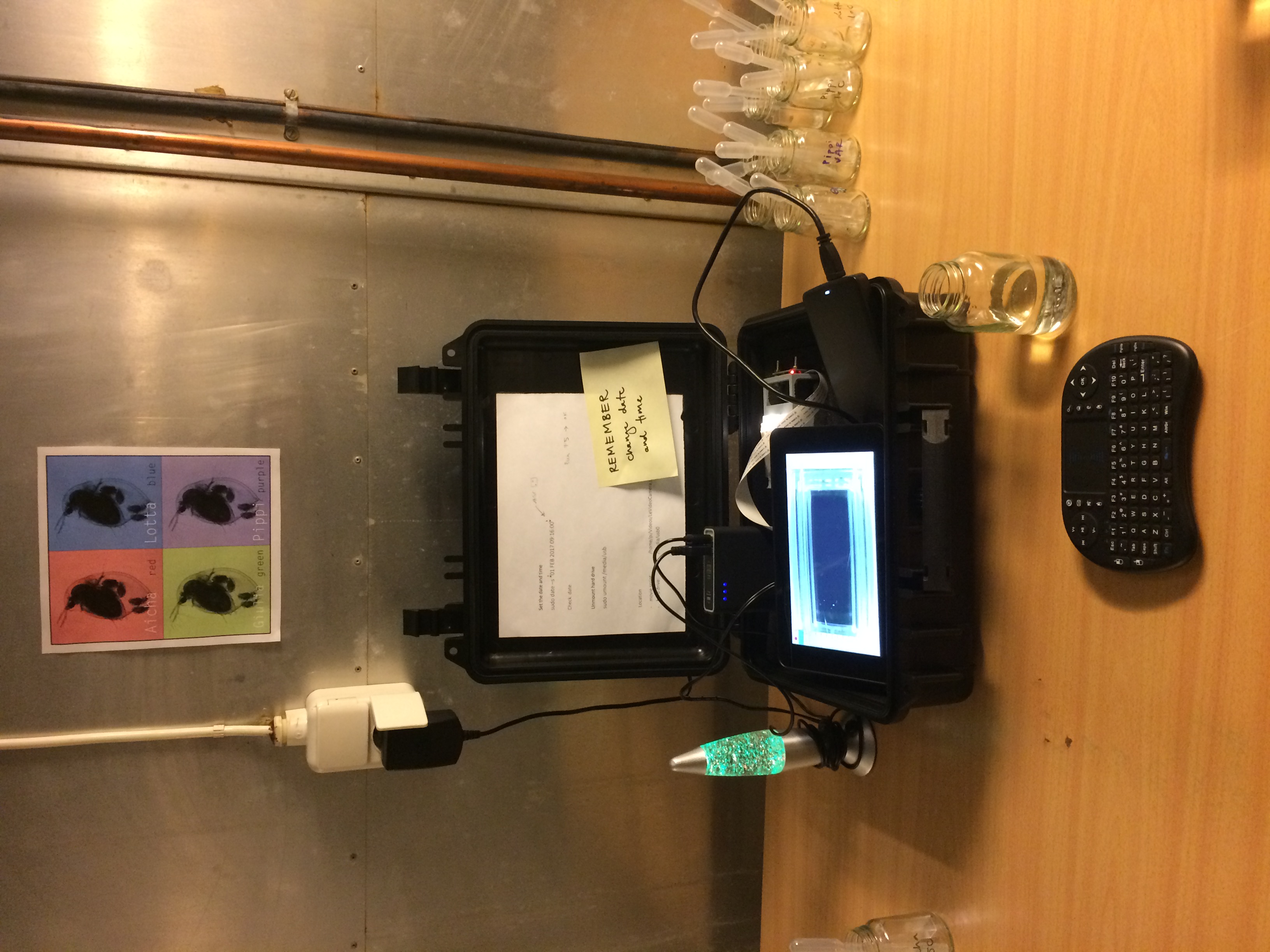

Supplement: Multimedia component 1 [file mmc1.zip › photoofsetup1.JPG]

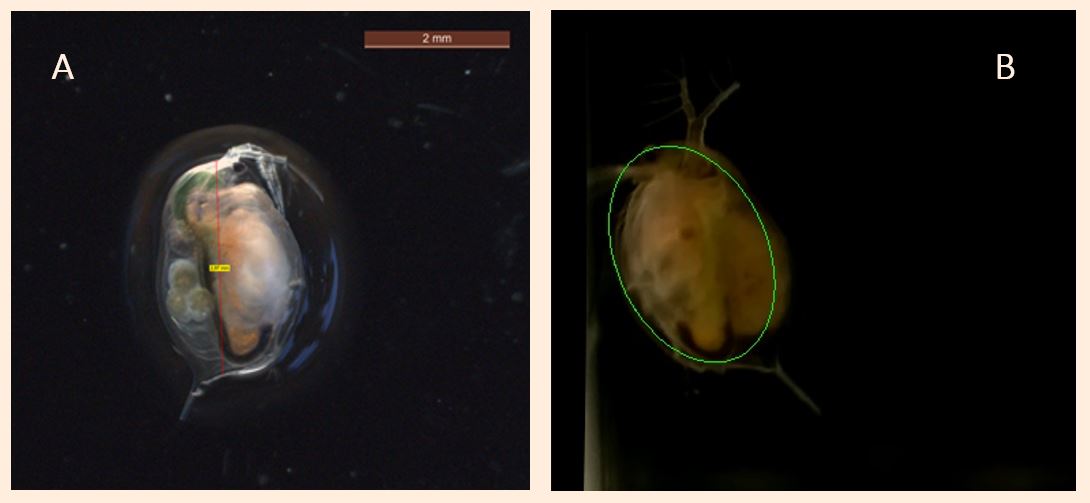

Supplement: Multimedia component 1 [file mmc1.zip › ManualMeasurementPics.JPG]
